# The effects of extra virgin olive oil or butter on cardiovascular biomarkers in European and Chinese males in the UK: A pilot randomised crossover trial

**DOI:** 10.1177/02601060231187516

**Published:** 2023-07-18

**Authors:** Fan Liang, Julie Young, Georgios Koutsidis, Jose Lara Gallegos

**Affiliations:** 1 Department of Applied Sciences, 373117 Faculty of Health and Life Sciences, Northumbria University, Newcastle upon Tyne, UK; 2 NUTRAN, Northumbria University, Newcastle upon Tyne, UK

**Keywords:** Extra virgin olive oil, cardiovascular risk factors, cardiovascular biomarkers, Caucasians, East Asians, randomised controlled trial

## Abstract

**Purpose:** No dietary interventional studies have reported the impact of olive oil on cardiovascular risk markers in groups of different ethnic origins. We report a pilot randomised controlled trial of an intervention supplementing extra virgin olive oil (EVOO) on markers of cardiovascular risk among East Asian Chinese, and European Caucasian individuals. **Methods:** A cross-over, randomised controlled dietary intervention for 2 weeks was undertaken. Thirty-two adults, healthy, individuals of Chinese and European origin took part in this study. 24-h ambulatory systolic and diastolic blood pressure (SBP, DBP), and blood lipids, were assessed. **Results:** Positive benefits of EVOO consumption were observed in all participants. Lower 24-h ambulatory SBP (−4.3 mmHg; *p* = 0.020), and day-time SBP (5.528 mmHg; *p* = 0.008), night-time DBP (−3.784 mmHg; *p* = 0.008) and night-time MAP (−3.747 mmHg; *p* = 0.007) were seen in all participants independently of covariates. In addition, total cholesterol (7.9 mg/dL; *p* = 0.017) and LDL-cholesterol (6.5 mg/dL; *p* = 0.028) were increased with butter but not with olive oil. No significant difference in other cardiovascular risk factors were observed. However, differences were observed between Caucasians and East Asian individuals in the absence of significant differences in lifestyle. **Conclusion:** This pilot study suggests that consumption of EVOO should be advocated as a healthier dietary fat and recommended to replace butter as a dietary strategy to improve cardiovascular health in both Caucasians and East Asian individuals.

## Introduction

Epidemiological and interventional studies have highlighted the health benefits of olive oil, particularly extra virgin olive oil (EVOO), or dietary patterns including olive oil such as the Mediterranean diet (Med-Diet). Systematic reviews of the epidemiological evidence indicate reductions in the risk of all-cause mortality (11%), cardiovascular mortality (12%), cardiovascular events (9%), and stroke (17%) (Schwingshackl and Hoffmann, 2014). These benefits are in part attributed to its high monounsaturated fatty acid (MUFA) content, especially oleic acid (C18:1) representing 55% to 83%. However, EVOO also contains phenolic compounds such as hydroxytyrosol and derivatives (oleuropein complex and tyrosol) which have also been associated with the prevention and progression of atherosclerosis (Marcelino et al., 2019). Furthermore, the presence of phytosterols (Kaur and Myrie, 2020), triterpenes (Sanchez-Rodriguez et al., 2019), different lipid classes particularly polar lipids (Karantonis et al., 2006), as well as antioxidants phenolic compounds or alpha-tocopherol have been involved in the health benefits of EVOO. A recent review summarises the effect of olive oil microconstituents in atherosclerosis (Antonopoulou and Demopoulos, 2023).

However, most trials evaluating EVOO, or the Med-Diet such as the PREDIMED study, have been carried out in Mediterranean countries (Estruch et al., 2018) or among white Caucasian individuals (Rasmussen et al., 1993; Thomsen et al., 1995). Little evidence exists on the health benefits or the acceptability of foods such as EVOO in racial/ethnic minority groups or in countries where EVOO is not commonly consumed (Sotos-Prieto and Mattei, 2018). Compared to white populations, individuals from ethnic minority groups are underrepresented in research, thus limiting the generalisability and potential public health impact of these interventions (Haughton et al., 2018). Caution against generalising results from predominantly white study populations to other racial/ethnic populations should therefore be adopted until further evidence is produced (Kris-Etherton et al., 2020). Countries such as the UK are increasingly diverse with ethnic minority groups representing 20–30% of the population and reflecting differences in dietary intake, dietary behaviours, and the burden of disease (Evandrou et al., 2016). The North East of England, where the current study took place, is characterised by high rates of obesity and CVD (Statistics on Obesity, Physical Activity and Diet, England, 2020), which account for 24% of all deaths (Cardiovascular disease prevention: state of the North East, 2019). Many of the risk factors for CVD including high blood pressure (BP), obesity, smoking, physical inactivity, excessive alcohol consumption, and a poor diet, are also common in Northeast England.

EVOO is associated with significant reductions of cardiovascular disease (CVD) and mortality (Guasch-Ferre et al., 2014) likely by reducing risk factors such as blood lipids and blood pressure (Ghobadi et al., 2019). Previous studies (Rasmussen et al., 1993) found that olive oil reduced BP after 3 weeks in 15 diabetic individuals, presumably Caucasians; only significant changes in day-time systolic (SBP) and diastolic blood pressure (DBP) were observed; no significant changes in night-time SBP or DBP were reported. In addition, Thomsen et al. (1995) reported significant changes in day-time SBP and DBP after 3 weeks of olive oil intake in 16 diabetic patients. Similarly, the EUROLIVE study reported significant reductions in HDL-cholesterol and total cholesterol/HDL ratio with high polyphenol olive oil (Covas et al., 2006). EUROLIVE also showed significant reductions on total-cholesterol and LDL-cholesterol (Martin-Pelaez et al., 2017). There is a need to determine if diverse populations, might benefit as well from foods such as EVOO. Some of this evidence has been systematically reviewed highlighting that diets enriched with olive oil versus diets rich in other fats, resulted in lower DBP, however, these results were characterised by high heterogeneity due to differences in the diet of control groups, characteristics of the populations studies and the type of olive oil (Zamora-Zamora et al., 2018). In spite of the evidence cited above, a recent consensus report of the III International Conference on Virgin Olive Oil and Health suggests that the effect of EVOO on plasma lipids is still contradictory and are not fully elucidated; therefore, it is recognised that more research is required in this area (Gaforio et al., 2019).

We report the results of a pilot randomised controlled trial (RCT) which evaluated the effects of an intervention supplementing EVOO or butter on cardiovascular biomarkers in European Caucasians and Chinese males living in the UK. The selection of these groups is based on the lack of evidence on Asian individuals and the fact that evidence suggests that risk for disease as well as mortality among these groups might be increased even at lower levels of body mass index (WHO, 2004). This study focused on men in order to minimise confounding originating from differences in dietary patterns and food choices between men and women (Beer-Borst et al., 2000; Wardle et al., 2004), and the impact of the menstrual cycle on the outcomes of interest (Schisterman et al., 2014).

## Materials and methods

The study was approved by the Northumbria University's Faculty of Health and Life Sciences Ethics Committee (Project ref: 10527). Informed signed consent was obtained from each participant. This study adheres to standard guidelines (Chan et al., 2013; Dwan et al., 2019). The protocol for this trial has been registered at Clinical Trials (https://clinicaltrials.gov/registration: NCT04187638).

### Trial design and randomisation

This study was a two-arms crossover RCT. Participants attended four study visits over a 6-week period.

Participants were randomised using computer-generated random integer generator software (http://www.randomization.com) in blocks of four, with a 1:1 ratio to ensure a balanced design between intervention order. This resulted in half of the participants starting the intervention taking EVOO and the other half starting with butter. The allocation sequence was concealed from the researcher collecting data (FL) using sequentially numbered, opaque, and sealed envelopes. Participants were unaware of the randomisation sequence; but given the nature of it, they were not blinded to the intervention.

### Eligibility criteria and study settings

Self-reported healthy, European and Chinese adult men (20–64 years of age), participated in this study. Participants were volunteers living around Newcastle upon Tyne, in Northeast England, UK. Participants of this study declared no issues in relation with the interventions and declared their commitment to comply with the interventions. All participants declared following an omnivorous diet with no interest or need for a special diet (e.g., weight loss, diabetes, cardiovascular risk factors). Exclusion criteria included consumption of dietary supplements; diagnosed with and/or taking medications for hypertension (>140/90 mmHg), diabetes, high blood cholesterol and heart problems; diagnosed lactose intolerance; taking omega-3 supplements regularly in the last 6 months, or if they reported being allergic to olive oil or butter.

### Sample size

Sample size calculations were performed for the primary outcome endpoint (SBP) using G*Power version 3.1.3 (Faul et al., 2009). A two tails *t*-test for two dependent means (matched pairs) was used. A statistical power of 0.80 and a 0.05 significance level, produced a sample of 12 in a crossover study to detect a difference of approximately 0.5 standard deviations in SBP (a difference of 6 mmHg between the responses to the intervention and control, based on previous studies). We aimed to recruit 15 male adults within each ethnic group in order to allow for a 20% drop-out rate.

### Interventions

In the intervention arm, participants were supplemented with 30 ml/day of EVOO for 2 weeks. The control arm received 30 g/day of butter for 2 weeks. EVOO and butter dose provided approximately 8–11% of energy from fat. These doses and duration were based on previous studies (Rasmussen et al., 1993; Thomsen et al., 1995; Madigan et al., 2000; Madigan et al., 2005) reporting that olive oil was effective in reducing BP in diabetic individuals.

Participants were instructed to replace their usual lunch meal with a meal including EVOO or butter plus one can of Heinz Cream of Vegetable Soup (400 g) or Heinz Potato and Leek soup (400 g), and to mix EVOO or butter within the soup. In addition, participants also were recommended to eat a piece of fruit (e.g., one banana: 105 kcal or one gala apple: 95 kcal) and one slice of white medium bread (94 kcal) so that the total calorie intake of the entire lunch meal was similar to the total calorie intake of a usual lunch meal, approximately 600 kcal (Supplemental Table 1). The choice of two different soups was established to improve compliance. Participants were provided with equal amounts of the soups and were recommended to alternate the consumption of these but were allowed to decide the order of consumption of these. The fats provided in this study were standard products available from supermarkets. These were purchased from Tesco – Tesco organic unsalted butter and Filippo Berio Organic EVOO. The nutritional composition of these is presented in Supplemental Table 1.

A 2-week time washout period was established. During the washout period, participants were asked not to consume olive oil or butter. Compliance and adverse effects with interventions were assessed by self-report using daily checklists. Self-reported daily checklist of EVOO showed a good acceptability and compliance with EVOO. No side effects of the intervention were reported regarding EVOO and butter consumption.

### Outcome measures and other procedures

The primary outcome measure of this study was ABP. Secondary outcomes included serum total-cholesterol, triglycerides, LDL-cholesterol, andf HDL-cholesterol analysed from the blood plasma samples. To minimise inter-observer variability, the same researcher (FL) carried out the clinical measurements. In addition, anthropometric measurements, determination of fatty acids in EVOO and butter were established. Prior to the intervention, they completed a 3-day dietary record (2 weekdays, 1 weekend day). They also recorded food intake during the first 2-week intervention period and were asked to repeat this dietary intake in the second arm of the study to match up the nutritional intake. They were asked to maintain their physical activity patterns throughout the whole study to limit body weight variability. Other measures such as dietary assessment methods included a 3-day dietary record and the 14-Item short Mediterranean Diet screener (Schröder et al., 2011). These are described in Appendix 1 of the supplementary material.

### Statistical analysis

All statistical analyses were carried out using IBM SPSS Statistics version 24. Data were evaluated for normality of distribution using the Shapiro–Wilk test. The general linear model (GLM) for repeated measures was used to compare within-subject treatment effects. Analysis involved mixed ANOVA with one within-subject factor (intervention) and one between-subject factor (ethnicity). Results (unadjusted and adjusted for covariates) are presented as means (or marginal means) ± SEM. Carry over effect and period effect were assessed using the statistical method for cross-over design by Wellek and Blettner (Wellek and Blettner, 2012). In the analysis undertaken in the full sample, we presented results as two models. Model 1 was adjustment for covariates including baseline and washout values, Model 2 was the most fully adjusted model, in which results were adjusted for covariates including baseline and post-washout values, ethnicity, BMI (kg/m^2^), age (years), intervention order sequence (whether EVOO or butter was consumed first), Mediterranean diet score, sodium (mg/day) intake, and energy intake (kcals/day) value. Paired *t*-tests were used to compare changes from baseline. Analysis was run on the whole sample, as well as grouped by ethnicity: A *p-*value less than 0.05 is assumed as significant.

## Results

**Figure 1.**
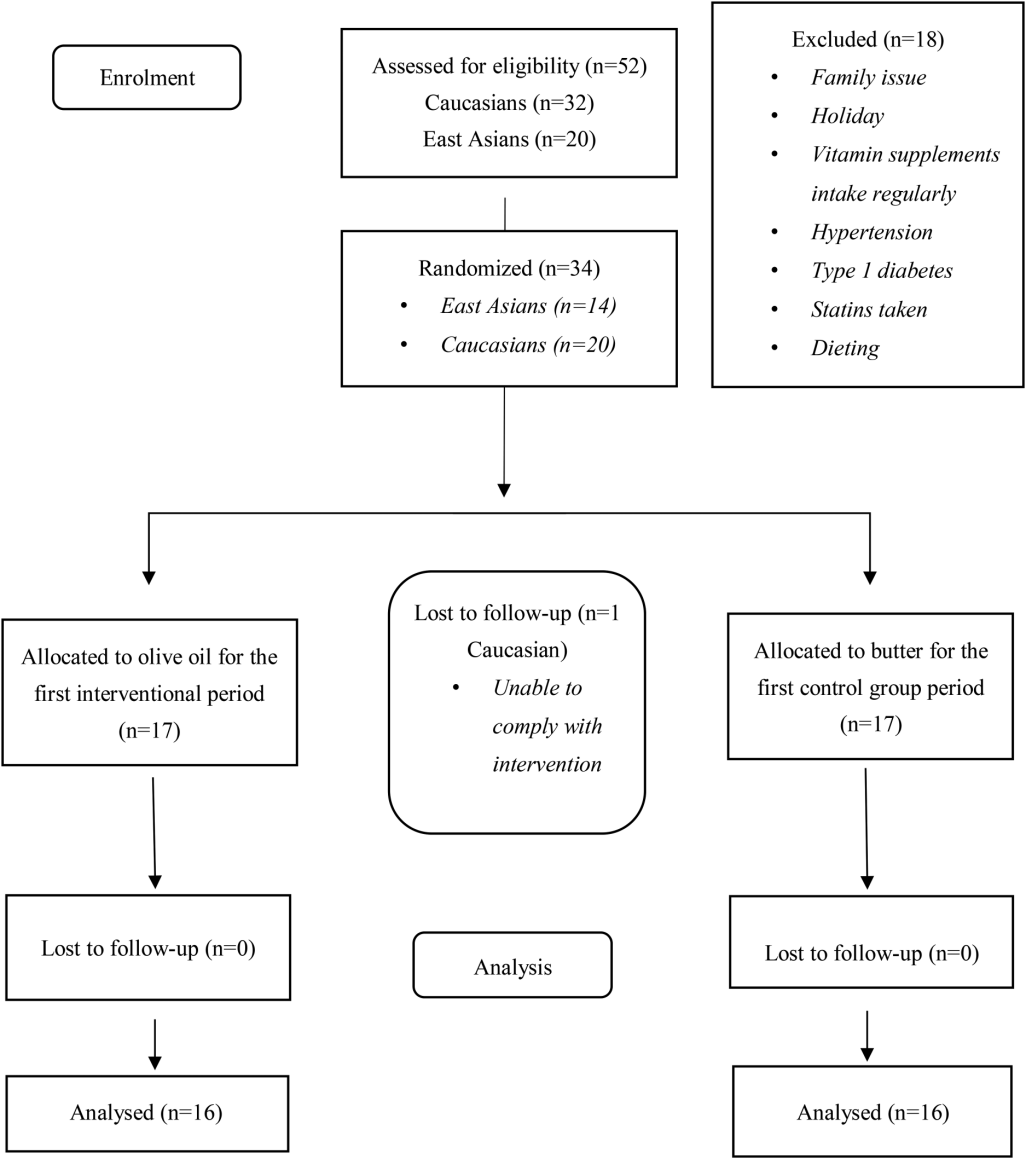
Consolidated standards of reporting trials (CONSORT) flow diagram for reporting of the participants’ recruitment and follow-up of the crossover human clinical trial.

In total 32 participants completed the intervention study (Figure 1). Participants reported 100% compliance with intervention meals. Research participants reported an overall good satisfaction with their experience in this study. In addition, no participants reported negative adverse effects. At the end of the study, all but one Caucasian participant reported they would continue using EVOO after taking part in the study.

### Participants characteristics

Participants of this study were on average mature adults, age ranged from 20 to 64 years old, of normal weight, normal BP, and body composition. The baseline characteristics of participants are presented for the full sample size as well as when these were divided according to their ethnicity into Caucasians (*n* = 18) and East Asians (*n* = 14). Overall, no significant differences were observed between groups at baseline on any of the variables presented in Table 1.

**Table 1.** Participants baseline characteristics (*n* = 32).

Baseline characteristics	All participants (*n* = 32)		Caucasians (*n* = 18)		East Asians (*n* = 14)		Mean difference (Caucasians − East Asians)
	Mean	SEM	Mean	SEM	Mean	SEM	
Age (year)	31.22	1.83	33.72	2.97	28.00	1.39	5.72
BMI (kg/m^2^)	24.11	0.66	24.51	0.82	23.59	1.10	0.92
Resting SBP (mmHg)	117.46	1.76	120.69	2.08	115.84	2.86	4.85
Resting DBP (mmHg)	68.48	1.80	68.26	2.23	68.76	3.04	−0.5
Resting HR (bpm)	68.23	1.87	67.39	2.74	69.30	2.50	−1.91
24-h SBP (mmHg)	119.63	1.37	120.69	2.08	118.26	1.66	2.43
24-h DBP (mmHg)	69.28	1.36	68.74	1.90	69.97	1.96	−1.23
24-h MAP (mmHg)	86.01	1.21	85.93	1.80	86.12	1.60	−0.19
Day-time ABP						
SBP (mmHg)	126.01	1.51	128.9	2.13	122.3	1.70	6.6
DBP (mmHg)	71.90	1.36	73.12	1.92	70.32	1.90	2.8
MAP (mmHg)	89.79	1.17	91.36	1.66	87.78	1.51	3.58
Night-time ABP						
SBP (mmHg)	113.34	1.64	114.93	2.48	111.29	1.92	3.64
DBP (mmHg)	64.57	1.17	64.81	1.96	64.27	1.02	0.11
MAP (mmHg)	80.88	1.20	81.43	2.00	80.16	1.07	1.27
Body composition						
Grip strength mean (kg)	39.4	1.20	39.53	1.90	39.24	1.34	0.29
Grip strength max (kg)	41.74	1.30	41.71	2.07	41.79	1.41	−0.08
Body fat (%)	16.31	0.96	16.70	1.31	15.82	1.45	0.88
Body fat (kg)	12.55	1.04	13.25	1.47	11.65	1.50	1.6
Lean (%)	82.14	1.34	81.99	1.68	82.34	2.25	−0.35
Lean (kg)	61.6	1.20	62.58	1.57	60.34	1.87	2.24
Blood-borne biomarkers						
TC (mg/dL)	169.8	5.8	163.6	9.7	173.6	7.3	10.1
HDL-C (mg/dL)	51.4	2.3	51.8	3.1	51.0	4.3	0.8
LDL-C (mg/dL)	101.7	5.4	95.9	9.3	105.2	6.2	9.3
Triglycerides (mg/dL)	81.5	6.2	78.8	8.0	79.7	8.0	0.9

SBP, systolic blood pressure; DBP, diastolic blood pressure; MAP, mean arterial pressure.

### Nutritional intakes

The nutritional intake of participants, as estimated from 3-day dietary diaries, is presented in Supplemental Table 2a. Supplemental Table 2a showed that there were no statistically significant differences in nutritional intakes of Caucasians and East Asian participants. In relation with habitual intakes of foods part of this intervention, four Caucasians reported consuming small amounts of olive oil (<10 ml) once during the 3-day of food records, while no East Asian reported consuming it; in relation with butter, seven Caucasians reported consuming butter (average of 15 g) twice during the 3-day recording period. Slight differences were observed in relation to food choices or eating out habits with the East Asians reporting foods characteristic of the Chinese traditional diet and a more frequent habit to eat out (Supplemental Table 2b). In addition, when using a short screener questionnaire to assess adherence to the Mediterranean diet (Supplemental Table 2c) none of the groups taking part in this study reported consuming EVOO or butter in amounts close to the dose or frequency used in this study.

### Anthropometrics

The weight, BMI, handgrip strength, and body composition of the participants, did not differ significantly between baseline, washout period and after consumption of EVOO and butter (Supplemental Table 3). These are suggestive of good compliance with instructions to not change lifestyle habits (diet, physical activity) during the intervention.

### Impact of intervention on outcome measures

#### Resting BP and 24-h ABP

Table 2 presents the baseline and post-intervention results by period (period 1 before and period 2 after washout), by dietary intervention (EVOO and Butter) for the two ethnic groups (Caucasians and Asians). No indication of carryover effect was observed.

**Table 2.** Ambulatory blood pressure by period, dietary intervention, and ethnic group

	Period 1								Period 2							
	EVOO				Butter				EVOO				Butter			
	Baseline		Post		Baseline		Post		Baseline		Post		Baseline		Post	
	Mean	SEM	Mean	SEM	Mean	SEM	Mean	SEM	Mean	SEM	Mean	SEM	Mean	SEM	Mean	SEM
Caucasians																
Day-time SBP (mmHg)	127.5	2.5	129.6	2.2	130.3	3.5	130.6	3.2	126.9	3.0	125.5	2.8	129.5	3.7	128.7	2.7
Day-time DBP (mmHg)	71.5	1.6	74.8	1.7	74.7	3.6	74.5	3.5	71.4	2.4	70.6	2.4	77.6	4.6	75.0	3.1
Night-time SBP (mmHg)	114.3	3.9	116.1	3.2	115.5	3.2	114.7	3.9	113.5	3.4	113.2	3.6	116.9	4.5	114.0	2.3
Night-time DBP (mmHg)	62.9	1.7	63.8	242	66.7	3.5	63.5	3.3	62.2	2.1	65.7	2.8	69.3	4.8	64.5	3.1
Asians																
Day-time SBP (mmHg)	120.9	2.1	116.7	2.1	123.7	2.7	123.6	2.7	122.5	1.8	122.4	3.2	121.7	3.0	126.4	4.1
Day-time DBP (mmHg)	68.3	2.0	68.4	2.6	72.4	3.2	75.0	3.0	73.1	1.9	71.3	1.6	74.5	3.7	75.9	4.7
Night-time SBP (mmHg)	110.9	2.8	104.4	1.5	111.7	2.8	113.7	2.7	108.5	1.9	109.6	2.5	113.5	2.1	115.5	4.3
Night-time DBP (mmHg)	64.1	1.9	58.9	1.0	64.4	0.9	66.0	1.2	65.3	1.3	66.7	1.3	68.6	2.8	69.6	4.0

Table 3 presents the results for resting and ABP for the whole sample. Results showed statistically significant differences on 24-h SBP, day-time SBP, night-time DBP and night-time MAP. Overall, after EVOO consumption, the 24-h SBP was significantly lower by 4.3 mmHg (*p* = 0.014) with adjustment for baseline and washout period in Model 1, and 4.3 mmHg (*p* = 0.020) with further adjustment in Model 2 (Table 3). In addition, the day-time SBP was significantly lower by 5.544 mmHg (*p* = 0.005) in Model 1 and 5.5 mmHg (*p* = 0.008) in Model 2, respectively (Table 3). Finally, after EVOO consumption, the night-time DBP was significantly lower by 3.7 mmHg (*p* = 0.030) in Model 1 and 3.8 mmHg (*p* = 0.008) in Model 2, respectively, while the night-time MAP was significantly lower by 3.7 mmHg (*p* = 0.026) in Model 1 and 3.74 mmHg (*p* = 0.007) in Model 2, respectively (Table 3).

**Table 3.** Effects of EVOO and butter on blood pressure in all participants (*n* = 32).

	Model 1						Model 2					
Variable	*N*	EVOO		Butter		*p*	EVOO		Butter		*p*
		LSM	SEM	LSM	SEM		LSM	SEM	LSM	SEM	
Resting SBP (mmHg)	32	116.87	1.57	118.43	1.33	0.344	117.00	1.62	118.57	1.19	0.362
Resting DBP (mmHg)	32	69.45	1.25	68.32	1.30	0.491	69.54	1.27	68.33	1.37	0.466
Resting HR	32	72.72	1.80	72.41	1.40	0.880	72.63	1.77	72.40	1.46	0.911
24-h ABP							
24-h SBP (mmHg)	32	117.65	1.19	121.96	1.31	*0*.*014*	117.64	1.25	121.98	1.35	*0*.*020*
24-h DBP (mmHg)	32	68.53	1.22	70.64	1.05	0.180	68.53	1.26	70.75	0.80	0.087
24-h MAP (mmHg)	32	85.39	1.06	87.91	1.02	0.072	85.39	1.12	88.01	0.84	*0*.*036*
Day-time ABP							
SBP (mmHg)	32	122.75	1.51	128.30	1.27	*0*.*005*	122.82	1.48	128.35	1.31	*0*.*008*
DBP (mmHg)	32	72.58	1.36	74.33	1.34	0.342	72.62	1.40	74.52	0.97	0.275
MAP (mmHg)	32	89.82	1.28	92.35	1.13	0.125	89.86	1.27	92.48	0.91	0.111
Night-time ABP							
SBP (mmHg)	32	111.31	1.57	114.86	1.73	0.077	111.37	1.58	114.97	1.57	0.067
DBP (mmHg)	32	63.16	1.28	66.86	1.60	*0*.*030*	63.25	1.22	67.03	1.39	*0*.*008*
MAP (mmHg)	32	79.61	1.20	83.27	1.59	*0*.*026*	79.69	1.21	83.43	1.32	*0*.*007*

Model 1 = Estimated from repeated measures ANOVA of the EVOO and Butter with baseline and washout value as covariate. Model 2 = Estimated from repeated measures ANOVA of the EVOO and Butter with baseline, washout, ethnicity BMI, age, Mediterranean diet score, intervention order sequence, sodium intake and energy intake value as covariate. LSM = least squared means (marginal means).

Results comparing the effect of interventions on resting and ABP only on Caucasian individuals are shown in Supplemental Table 4. Findings indicate that after EVOO night-time DBP and night-time MAP was significantly lower by 4.1 mmHg (*p* = 0.047) and 3.9 mmHg (*p* = 0.06) in Model 2. Supplemental Table 5 presents the results for only East Asian participants; 24-h SBP was significantly lower after EVOO by 5.9 mmHg (*p* = 0.026), 24-h MAP was significantly lower 3.8 mmHg (*p* = 0.038), day-time SBP was significantly lower by 8.2 mmHg (*p* = 0.032), and day-time MAP was significantly lower by 5.2 mmHg (*p* = 0.052) in Model 2 after EVOO intake too (Supplemental Table 5).

Supplemental Table 6 shows the values for dipping (day-time BP minus night-time BP). Results showed differences between Caucasians and East Asians, with Caucasians showing higher dip than Caucasians for both SBP and DBP. Caucasians’ dip in SBP and DBP was lower after consuming Butter than after consuming EVOO; while in East Asians a different pattern was observed with a lower dip in SBP after EVOO and only a minimal variation in DBP.

#### Blood-borne biomarkers

Table 4 presents the baseline and post-intervention blood lipids results by period, dietary intervention, and ethnic group. No indication of carryover effect was observed.

**Table 4.** Blood lipids by period, dietary intervention, and ethnic group

	Period 1								Period 2							
	EVOO				Butter				EVOO				Butter			
	Baseline		Post		Baseline		Post		Baseline		Post		Baseline		Post	
	Mean	SEM	Mean	SEM	Mean	SEM	Mean	SEM	Mean	SEM	Mean	SEM	Mean	SEM	Mean	SEM
Caucasians																
Total Cholesterol (mg/dL)	152.5	12.1	146.1	8.9	163.3	4.9	168.0	5.3	170.1	11.3	169.3	7.1	146.1	8.5	156.8	8.4
HDL-C (mg/dL)	54.1	3.6	54.6	3.9	49.8	4.3	49.9	4.7	49.4	4.7	50.3	4.9	54.1	4.9	55.9	4.3
LDL-C (mg/dL)	84.6	12.7	77.8	10.2	98.0	3.8	102.3	4.1	107.0	13.1	104.8	10.3	75.2	9.8	85.9	10.6
Triglycerides (mg/dL)	67.9	7.2	63.0	10.1	84.6	10.9	90.5	8.6	90.5	12.9	90.5	6.5	85.6	15.6	73.8	10.0
Asians																
Total Cholesterol (mg/dL)	185.6	6.7	180.1	8.3	169.6	11.1	175.1	14.8	162.4	10.7	159.1	10.9	183.4	7.5	190.0	7.4
HDL-C (mg/dL)	56.3	7.1	53.6	6.3	44.2	4.6	44.8	4.6	43.6	3.6	42.0	5.3	52.5	5.9	56.9	7.1
LDL-C (mg/dL)	112.7	5.2	101.1	5.1	103.3	9.4	106.6	13.2	97.2	10.2	90.0	9.6	103.9	8.7	108.8	7.1
Triglycerides (mg/dL)	93.6	10.6	96.2	11.8	106.2	12.9	113.9	18.4	78.4	13.4	79.7	14.7	102.5	10.6	126.5	14.8

Results in the total sample showed that after EVOO, statistically significant difference for total-cholesterol was observed 8.15 (*p* = 0.008) in Model 1 and 7.9 (*p* = 0.017) in Model 2 (Table 5). After EVOO consumption, LDL-C was significantly lower by 6.7 mg/dL (*p* = 0.018) in Model 1 and 6.45 mg/dL (*p* = 0.028) in Model 2, respectively, in the group of all participants (Table 5).

**Table 5.** Effects of EVOO and butter on blood-borne biomarkers in all participants (*n* = 32).

		Model 1						Model 2				
Variable	*N*	EVOO		Butter		*p*	EVOO			Butter		*p*
		LSM	SEM	LSM	SEM		LSM		SEM	SEM	SEM	
TC (mg/dL)	32	173.5	3.1	165.5	3.5	*0*.*008*	165.5		3.5	173.6	3.5	*0*.*017*
HDL (mg/dL)	32	50.4	0.8	51.8	1.2	0.153	50.3		0.8	51.8	1.2	0.168
LDL (mg/dL)	32	95.5	1.5	102.1	2.3	*0*.*018*	95.9		1.5	102.1	2.3	*0*.*028*
Triglycerides (mg/d L)	32	102.5	8.0	125.8	24.8	0.243	126.7		23.0	103.6	7.1	0.229

Model 1: Estimated from repeated measures ANOVA of EVOO adjustment with baseline, washout value as covariate. Model 2: Estimated from repeated measures ANOVA of EVOO adjustment with baseline, washout, ethnicity, intervention order sequence, BMI, age, Mediterranean diet score, energy intake, PUFAs, MUFAs, trans fatty acids, saturated fatty acids, and dietary cholesterol value as covariate. LSM = least squared means (marginal means).

When only Caucasian individuals were analysed (Supplemental Table 7), after EVOO a significantly lower LDL-C by 8.4 mg/dL (*p* = 0.046) with adjustments in Model 1, and 8.39 mg/dL (*p* = 0.041) with further adjustment in Model 2 (Supplemental Table 7) were observed. For non-HDL-C, after EVOO non-HDL-C was significantly lower by 10.1 mg/dL (*p* = 0.048) in Model 1.

In the group of East Asians (Supplemental Table 8), TC was significantly lower by 8.0 mg/dL (*p* = 0.044) in Model 1, respectively. However, after covariate adjustments in Model 2 this result was not statistically significant.

In Supplemental Table 9, the difference of 24-h day-time SBP between EVOO intake before and after is shown to be associated with fat intake (*p* = 0.004) while the difference of 24-h night-time DBP between EVOO intake before and after is associated with sodium intake (*p* = 0.045). There was no correlation between TC, LDL, and dietary fats.

#### Determination of fatty acids in EVOO and butter

Supplemental Table 10 shows the fatty acid composition of the EVOO and butter used in this intervention. Butter was 65% saturated fatty acids; the main fatty acids were palmitic acid C16:0 (31%), Oleic acid C18:1n-9 (25%), and stearic acid C18:0 (14.5%). EVOO was 16% saturated fatty acids; the main fatty acids were Oleic acid C18:1n-9 (73%), palmitic acid C16:0 (13%), and linoleic acid C18:2n-6 (6.8%). These profiles are very similar to those reported in other studies.

## Discussion

### Principal findings

**Figure 2.**
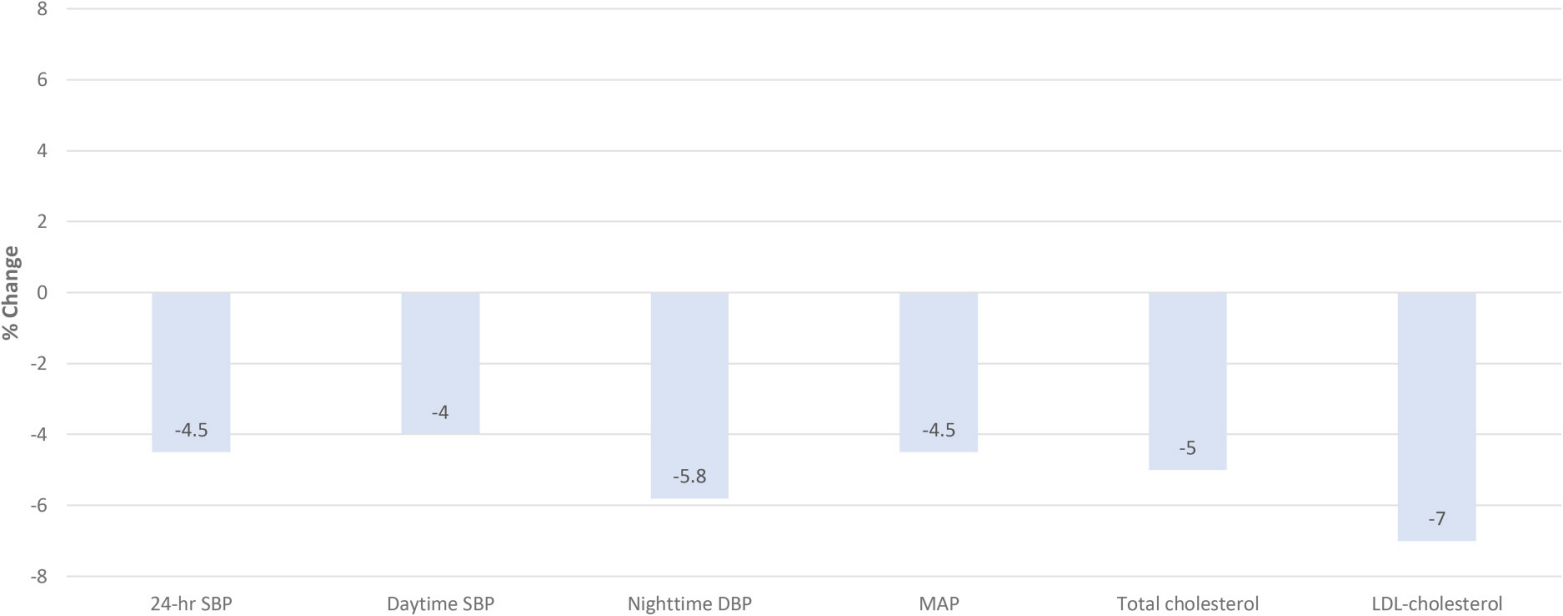
Percentage change in cardiovascular risk factors. SBP, systolic blood pressure; DBP, diastolic blood pressure; MAP, mean arterial pressure.

Our analysis of the whole sample combined, showed that consumption of 30 ml of EVOO, instead of butter, over a 2-week period was associated with positive effect on 24-h SBP (−4.5%), day-time SBP (−4.0%), night-time DBP (−5.8%), and MAP (−4.5%). In, addition a lower TC (−5.0%), LDL-C (−7.0%) was observed when consuming EVOO instead of butter, even after adjustment for covariates (Figure 2). However, it is important to mention that when analysing the sample by ethnic group, important differences were observed. Asian individuals seemed more responsive to the interventions tested in this study with some significant results on 24-h SBP and MAP, day-time SBP and MAP; while the Caucasians showed a significantly lower night-time DBP and a non-significant trend towards lower nigh-time MAP. Similarly, Asians showed a lower total cholesterol, while Caucasians showed lower LDL and non-HDL cholesterol associated with EVOO. Such differences in the response to interventions did not seem to be associated with dietary practices or changes in body fat and BMI. These findings warrant further investigation on the impact of ethnic and genetic background on dietary intervention.

The results of this study might be important for practicing dietitians and policymakers. These results are significant given the fact that high BP remains ranked first among the leading risk factor for death and disability (Collaborators, 2020). The combination of BP and cholesterol improvements are seen after consuming EVOO instead of butter, if sustained over time, are likely to reduce the risk of CVDs independently of ethnic background. This is particularly important for East Asian individuals and populations whose cardiovascular risk may be present at lower BMIs. Nonetheless, the most practical application of this pilot study is it provides data on the variability of the main outcomes enabling estimation of the sample size for a definitive trial.

### Scientific analysis of findings

The study of the health benefit of dietary interventions in ethnic minorities living in developed countries is still limited (Haughton et al., 2018), and the present study adds to the evidence.

Evidence suggests that different types of fat, rather than total fat intake, play a significant role in cardiovascular health (Forouhi et al., 2018). In line with our results, previous studies of a duration like ours and using similar doses of EVOO (Rasmussen et al., 1993; Thomsen et al., 1995; Madigan et al., 2000; Madigan et al., 2005) reported a reduced BP in diabetic individuals. In addition, the PREDIMED study reported significant long-term reductions in 24-h, day-time, and night-time SBP and DBP after 1 year with a Mediterranean diet plus EVOO in male and female (Domenech et al., 2014). Other studies assessing resting BP have also reported, in healthy men from non-Mediterranean countries, a 3% reduction in SBP after 3 weeks of 25 ml/d of olive oil (Bondia-Pons et al., 2007). In relation to blood markers, positive effects of olive oil on blood lipids have been previously reported in a predominantly white Caucasians sample in the UK; 50 g/d of olive oil for 4 weeks significantly lowered LDL-C in comparison to butter (Khaw et al., 2018).

Contrasting with the evidence involving white Caucasians, intervention studies evaluating the effects of olive oil on BP among East Asians are lacking. Epidemiological studies such as the international study of macro/micronutrients and blood pressure (Miura et al., 2008; Miura et al., 2013) reported that MUFAs were inversely associated with DBP among 17 population samples in China, Japan, UK, and USA. Similarly, the Korean Genome and Epidemiology Study (Lee et al., 2019), reported that high MUFAs intake was inversely associated with the risk of hypertension (OR = 0.49, 95% CI = 0.29–0.82). In addition, a recent RCT in middle-age male and female individuals in China, reported that olive oil consumption had no significant effect on total-, LDL-, HDL-cholesterol, or triacylglycerols (Sun et al., 2018). To our knowledge, there are no other interventional studies in East Asian individuals published in the scientific literature.

The cardiovascular beneficial effects of EVOO have been attributed to a complex array of components and properties including its high MUFAs content, the presence of phytosterols (Kaur and Myrie, 2020), triterpenes (Sanchez-Rodriguez et al., 2019), different lipid classes particularly polar lipids (Karantonis et al., 2006), as well as antioxidants phenolic compounds or alpha-tocopherol. However different MUFAs seem to show differential effects. A study in rats consuming a diet enriched with oleic acid, the main fatty acid present in EVOO, showed a marked and significant decrease in SBP in rats (Berrougui et al., 2015); the authors presented evidence for an increase of oleic acid in cell membranes. Two human studies, 1 in 12 healthy women (RuizGutierrez et al., 1997) and 1 in 16 hypertensive women (RuizGutierrez et al., 1996), showed a significant reduction in BP associated with EVOO but not with high-oleic sunflower oil. In addition, results from the OmniHeart study showed that a diet rich in monounsaturated fat consumed for 6 weeks significantly reduced SBP in both prehypertensive and hypertensive individuals (Appel et al., 2005).

There is also evidence supporting the role of EVOO polyphenols in lowering BP. A 2-month crossover RCT in young women with mild hypertension reported that a diet containing polyphenol-rich olive oil (564 mg/kg of polyphenols), compared with a polyphenol-free olive oil, lead to a significant (*p* < 0.01) decrease of 7.91 mmHg in SBP and 6.65 mmHg of DBP (Moreno-Luna et al., 2012). In addition, results from the EUROLIVE study in adult men, showed that 25 ml of a high polyphenol EVOO (366 mg/kg) versus a low polyphenols olive oil (2.7 mg/kg), reduced SBP but not DBP (Martin-Pelaez et al., 2017).

In relation with the effect of olive oil polyphenols on blood lipids, the EUROLIVE study reported significant reductions in HDL cholesterol and total cholesterol/HDL ratio with high polyphenol olive oil (Covas et al., 2006). Another from EUROLIVE reported significant reductions on total-cholesterol and LDL-cholesterol (Martin-Pelaez et al., 2017). In addition, a study in 90 adult men and women in Spain reported that a Mediterranean diet combined with virgin olive oil significantly reduced LDL-cholesterol at 3 months, when compared with groups following a Mediterranean diet plus washed olive oil, or with a control group. It is likely that both oleic acid and phenolic compounds in the EVOO evaluated in the present study, contributed to observed changes in ABP and blood lipids.

Epidemiological evidence suggests that the effects observed in the present study are likely to have significant health implications if sustained in the long term. In relation with the observed reductions in BP, some evidence indicates that a decrease in SBP of ≥5 mmHg could decrease the risk of stroke mortality by 13–14% (Reboldi et al., 2011) and cardiovascular diseases mortality by 9% (Chobanian et al., 2003). In relation with the observed reductions in blood lipids, the clinical significance of the reductions in total- and LDL-cholesterol are likely to have a lesser impact. Although a linear relationship between cholesterol reductions and risk reductions has been shown (Wang et al., 2020), greater reductions seem to be required to achieve benefits; a net reduction in total serum cholesterol of 10% translates to an expected 15% reduction in CHD mortality (Gould et al., 1998), while a reduction of 1.0 mmol/L in LDL-cholesterol would translate into reductions of 12% in vascular mortality (attributable to reductions of 20% in CHD deaths and 8% in cardiac deaths; Mihaylova et al., 2012). Interventional and epidemiological data supports these estimates. Epidemiological observational studies support these estimates. Analysis from the PREDIMED study involving individuals at high cardiovascular risk in Spain (Guasch-Ferre et al., 2014), reported that after 4.8 years follow-up those in highest energy-adjusted tertile of baseline total olive oil had a reduced the risk of CVD by 35% (HR: 0.65; 95% CI: 0.47 to 0.89), in comparison higher EVOO consumption reduced the risk of CVD by 39% (HR: 0.61; 95% CI: 0.44 to 0.85). In addition, a higher baseline total olive oil consumption was associated with 48% lower risk of cardiovascular mortality (HR: 0.52; 95% CI: 0.29 to 0.93). It was reported that for each 10 g/d increase in extra-virgin olive oil consumption were associated with 10% lower risk for cardiovascular disease and 7% lower risk for mortality. Similarly, analysis from the “nurses’ health study” and the “health professionals follow up study,” showed that after 24 years of follow-up those with higher olive oil intake (>1/2 tablespoon/d or > 7 g/d) had 14% lower risk of total CVD (pooled HR 0.86 [95% CI 0.79, 0.94]) and 18% lower risk of CHD (pooled HR 0.82 [85% CI 0.7 to 0.91]). This study also estimated that replacing 5 g of margarine, butter, mayonnaise, or dairy fat with the equivalent amount of olive oil was associated with 5–7% lower risk of total CVD and CHD (Guasch-Ferre et al., 2020). The potential impact of olive oil in reducing cardiovascular risk factors and the risk for morbidity and mortality is important given that high BP and LDL-cholesterol remain the top 10 leading risk factors for disease, disability and mortality (Collaborators, 2020), and primary prevention remains an important public health approach to tackle these (Collaboration, 2021).

Different mechanisms for the hypotensive effects of olive oil, and its components, have been proposed (Massaro et al., 2020). The hypotensive properties of phenolic acids may be related to mechanisms involving NO-mediated vasodilatory effects, the attenuation of oxidative stress (reactive oxygen species) by reducing NAD(P)H-dependent (nicotinamide adenine dinucleotide phosphate) super-oxide production, and the interaction with the renin–angiotensin aldosterone system by inhibiting angiotensin-converting enzyme activity (Massaro et al., 2020). The hypotensive effects of Oleic acid, on the other hand, incorporates into the cell membranes of blood vessels where it likely makes the cells more receptive to signals that reduce BP. Alterations in the structure and function of the cell membrane have been associated with hypertension (Massaro et al., 2020). A study in rats showed that the increase in oleic acid within cellular phospholipids downregulates the expression of a series of G protein-coupled receptors, including the adrenoceptor alpha 2A, the G protein subunit alpha i2, the G protein subunit alpha i3, the G protein subunit alpha 11, and phospholipase C beta, which inhibits adenylyl cyclase activity (Teres et al., 2008).

Other factors might have been involved in the results observed in the present study. It is well established that BP levels are higher during the day and lower at night (Mancia and Grassi, 2014). BP rises sharply in the morning after activation of the sympathetic nervous system on morning arousal. Awakening selectively stimulates the sympathoadrenal branch of the sympathetic nervous system and increases epinephrine levels. In addition, the natural circadian rhythm of BP presents a nocturnal decrease of 10–20% in BP, called the “dip” (Routledge and McFetridge-Durdle, 2007; Bloomfield and Park, 2015). The present study showed differences in dip between Caucasians and East Asians, with slightly greater values in Caucasians. In addition, in Caucasian individuals this decrease in SBP was slightly increased after EVOO. In contrast to increased sympathetic activity, parasympathetic activity is decreased in patients with hypertension, suggesting a sympathetic/parasympathetic imbalance. Some research suggests that absence of “dipping” may result from a decrease in parasympathetic function (Routledge and McFetridge-Durdle, 2007). Changes in the sympathetic and parasympathetic nervous systems might be important determinants of changes in night-time BP (Grassi et al., 2010). Other factors such as quality of sleep and global sleep patterns, not addressed in this study, have also been related to reduction in night-time BP (Thomas et al., 2020). Night-time BP is a predictor of risk for cardiovascular outcomes and mortality, independent of day-time BP (Hansen et al., 2011).

### Strengths

This study has several strengths. Firstly, the improvements observed in BP reported were documented using robust methodology such as 24-h ABP. Secondly, a high-completion rate was present with 94% of the sample completing this study; self-reported daily checklist of EVOO showed a good acceptability and compliance with both EVOO and butter intake. No side-effect of interventions was reported. The fact that BMI, body composition and handgrip strength remained unchanged during the whole clinical trial, supports the presence of good compliance with maintaining usual diet and physical activity patterns. Another strength of the present study is lack of evidence for carryover effects characteristics of crossover designs, which permitted the same participants to receive all interventions and thereby minimised interferences with confounding variables.

### Limitations

This study is, however, not without limitations. Firstly, this study was an “open-label” study that could not blind participants, a common situation in dietary interventions. The fact that 30 ml of EVOO and 30 g of butter are not equivalent might be questioned. Self-reporting of information is likely to be a limitation in dietary interventions, however, a good self-reported compliance together with changes in the outcomes of interest, suggest this is likely to have been minimised. In addition, the different dietary patterns between Caucasians and East Asians, with most British participants following a Westernised diet, is likely to confound effects of interventions and results (Leung and Stanner, 2011) . Our study only included men, and results cannot be generalised to women.

Finally, lack of data on the phenolic composition of EVOO and butter used in this study, does not allow the investigation of whether the positive benefits on cardiovascular risk factors found in this study were due to any, or a combination of these compounds.

## Conclusion

The findings of this pilot study suggest that consumption of EVOO for 2 weeks, instead of butter, is associated with significantly lower 24-h SBP including day-time SBP, night-time DBP and night-time MAP, as well as TC, LDL-C, in healthy male volunteers. However, differences were observed between Caucasians and East Asian individuals in the absence of significant differences in lifestyle. Thus, further research on this area is warranted, and the study of other ethnic groups should be encouraged. In conclusion, consumption of EVOO, instead of butter, could improve cardiovascular health of individuals, however, it is currently acknowledged that evidence on the effect of EVOO on plasma lipids is still controversial and further research is required before drawing solid conclusions.

## Supplemental Material

sj-docx-1-nah-10.1177_02601060231187516 - Supplemental material for The effects of extra virgin olive oil or butter on cardiovascular biomarkers in European and Chinese males in the UK: A pilot randomised crossover trial Supplemental material, sj-docx-1-nah-10.1177_02601060231187516 for The effects of extra virgin olive oil or butter on cardiovascular biomarkers in European and Chinese males in the UK: A pilot randomised crossover trial by Fan Liang, Julie Young, Georgios Koutsidis and Jose Lara Gallegos in Nutrition and Health
