# Supplementary material for: The effects of extra virgin olive oil or butter on cardiovascular biomarkers in European and Chinese males in the UK: A pilot randomised crossover trial
Source: Nutr Health. 2023 Jul 18;31(2):485–98. doi: 10.1177/02601060231187516 (PMC12174621; doi:10.1177/02601060231187516)
Supplement: sj-docx-1-nah-10.1177_02601060231187516 - Supplemental material for The effects of extra virgin olive oil or butter on cardiovascular biomarkers in European and Chinese males in the UK: A pilot randomised crossover trial [file sj-docx-1-nah-10.1177_02601060231187516.docx]

**The effects of extra virgin olive oil or butter on cardiovascular biomarkers in European and Chinese males in the UK: A randomized crossover trial**

**Authors and Affiliations: Fan Liang ^1^ , Julie Young ^1^, Georgios Koutsidis^1^, Jose Lara Gallegos^1,2,3^**

1. Department of Applied Sciences, Faculty of Health and Life Sciences, Northumbria University, Newcastle upon Tyne, UK; [fan.liang2292823574@gmail.com](mailto:fan.liang2292823574@gmail.com) (FL), [julie2young@northumbria.ac.uk](mailto:julie2young@northumbria.ac.uk) (JY), [georgios.koutsidis@northumbria.ac.uk](mailto:georgios.koutsidis@northumbria.ac.uk) (GK), [jose.lara@northumbria.ac.uk](mailto:jose.lara@northumbria.ac.uk) (JLG).
2. NUTRAN, Northumbria University, Newcastle upon Tyne, UK
3. Correspondence: [jose.lara@northumbria.ac.uk](mailto:jose.lara@northumbria.ac.uk)

***Appendix 1***

***2.5. Outcome measures and procedures***

*2.5.1. Resting BP and 24-hour ABP*

Resting BP was measured in a quiet room after participants rested for 15 minutes in a seated position with arm resting on a firm surface and feet flat on the floor, using a non-invasive digital automatic BP monitor (Carescape™ V100: GE Healthcare, UK). BP measurements were taken in the non-dominant upper arm in triplicate, and the average of the last two measurements was used for analyses.

A non-invasive ABP device (Medical 90217-1Q: Spacelabs, Inc. Richmond, Washington, USA) was utilized to record and measure 24-hour ABP. The monitor was programmed (ABP Report Management System version 3.0.3 Spacelabs, Inc. Richmond, Washington, USA) to inflate automatically, at 30-min intervals during 0800-2200 and 60-min intervals during 2200-0800, for a total period of 24-hours. Mean daytime and night-time BP were calculated based on measurements taken while participants were awake and asleep, respectively [19].

*2.5.2. Fasting blood sample collection and analysis*

Fasting blood was drawn into separate 6 mL BD Vacutainer® Heparin Tube tubes (Becton Dickinson) and centrifuged immediately for 15 min at 1500rpm. Plasma samples were immediately stored at -80 ℃ until analysis. Samples were analysed at the end of study by staff unaware of treatment allocation.

Blood lipids were measured at the integrated laboratory medicine at Freeman Hospital in Newcastle Upon Tyne. Plasma triglycerides were measured by an enzymatic, colorimetric method with glycerol phosphate oxidase and peroxidase (Trigl, Cobas Roche Diagnostics, Indianapolis, IN, USA). Total-cholesterol was obtained by an enzymatic, colorimetric method through the cholesterol esterase/cholesterol oxidase/peroxidase reaction (CHOL2, Cobas Roche Diagnostics, Indianapolis, IN, USA). The plasma concentration of HDL-cholesterol was determined by a homogeneous enzymatic colorimetric assay through the cholesterol esterase/cholesterol oxidase/peroxidase reaction (HDLC3, Cobas Roche Diagnostics, Indianapolis, IN, USA). LDL cholesterol was calculated using the Friedewald equation LDL = Total Cholesterol - HDL Cholesterol – (Triglycerides / 2.2). This equation is not valid if triglycerides are >4.5 mmol/L, but in the present study, no values were observed above such limit.

*2.5.3 Anthropometrics and body composition assessment - Bioelectrical impedance*

Measures of height (m) and weight (kg) were used to calculate BMI (kg/m2). A stadiometer was used to measure height to the nearest centimetre (cm).

Bioelectrical impedance analysis (BIA) was performed using a single frequency (50 kHz) device (Body stat 1500, Body stat Ltd; Isle of Man, UK). Measured in a supine position electrode pads were placed in the middle of the dorsal surface of the left hand and left foot; a second set of electrodes were placed between the distal prominence of the radius and the ulnar styloid and between the medial and lateral malleoli at the ankle.

*2.5.4. Handgrip strength*

Handgrip strength was measured using a CAMRY-EH101 hand dynamometer (range 0 to 90kg; accuracy 0.1 kg) (EH101; Camry, Guangdong Province, China). In a sitting position, participants hold the dynamometer in their dominant hand. The handle of the dynamometer was adjusted if required. Participants were instructed to squeeze the dynamometer with maximum effort and hold for approximately 5 seconds without other body movements[20]. Handgrip strength was assessed in triplicate and the average of all readings were taken as the final score.

*2.5.5. Dietary assessment and nutritional analysis*

Participants were asked to record their dietary intake for 3 non-consecutive days (i.e., type of foods, preparation and amount of food/drink consumed), two weekdays and one weekend day. The 3-day dietary record was estimated by providing guidance on the estimation of household measures, and food intake record from each participant allowed a nutritional analysis with the aid of the ‘‘Micro-diet’’ software (Micro-diet System, Version 6.4, Salford University, UK). In addition, a validated short screener questionnaire to assess adherence to a Mediterranean dietary pattern was used. This screener includes 14 questions evaluating the frequency of several foods and food groups (these are listed in supplementary table S2c).

*2.5.6 Determination of Fatty acid Methyl esters in food samples*

Approximately 25 mg of either EVOO or butter samples were accurately weighed, followed by the addition of 5 mL of hexane and 1 mL of 2N potassium hydroxide in methanol. All reagents were kept over anhydrous sodium sulphate. The samples were left to esterify overnight, the hexane phase recovered and subsequently analysed using an Agilent 6890N gas chromatograph coupled to an Agilent 6953N mass spectrometer. Fatty acid methyl esters were separated on a DB-23 capillary column (60 m (L) x 0.25 mm (D) x 0.25 um (FT). The samples (1 µL) were injected in split mode (100:1) at an injection port temperature of 250 °C, using helium as a carrier gas at a flow rate of 1 mL/min. The initial oven temperature was kept at 45 °C for 1 min, followed by a temperature ramp at 25 °C/min to 95 °C, held for 1 min, then at 15 °C/min to 190 °C held for 12 min, then at 3 °C/min to 250 °C held for 5 min. The Mass spectrometer was operated at the total ion mode (scanning range m/z 33-550). The transfer line was held at 250 °C while the quadrupole and ion source were kept at 180 °C and 230 °C respectively. Compound Identification and quantitation were performed using the CRM47885 FAME reference standard (37 FAME reference compound mix) (Supelco). All analysis were performed in triplicate.

**Supplementary Materials**

**Table S1. Nutrient Content 30 ml of extra virgin olive oil, 30 g of butter, 400g of soup respectively; Data obtained from USDA Food Composition Databases and Tesco product description.**

| **Nutrient (unit)** | **EVOO** | **Butter** | **Heinz Vegetable**  **Soup** | **Heinz Potato and**  **Leek soup** |
| --- | --- | --- | --- | --- |
| **Total Fat (g)** | 27.39 | 24.6 | 3.4 | 7.4 |
| **Saturated Fat (g)** | 4.65 | 15.6 | 0.2 | 4.4 |
| **Trans Fat (g)** | 0 | 0 | 0 | 0 |
| **Polyunsaturated Fat (g)** | 3.21 | 0 | 0 | 0 |
| **Monounsaturated Fat (g)** | 19.53 | 0 | 0 | 0 |
| **Cholesterol (mg)** | 0 | 0 | 0 | 0 |
| **Iron (mg)** | 0.07 (0) | 0 | 0 | 0 |
| **Energy (kJ)** | 1013.4 kJ | 918.6 kJ | 792 kJ | 832 kJ |
| **Energy (kcal)** | 242.2 kcal | 219.5 kcal | 189.3 kcal | 198.9 kcal |
| **Carbohydrate (g)** | 0 | 0.18 | 33.2 | 28.6 |
| **Sugars (g)** | 0 | 0.18 | 13 | 0 |
| **Fibre (g)** | 0 | 0 | 3.6 | 2.2 |
| **Salt (g)** | 0 | 0.03 | 2.4 | 2.4 |
| **Protein (g)** | 0 | 0.18 | 4.4 | 3.2 |

**Table S2a. Daily intakes of nutrients by Caucasians and East Asians from 3-d dietary records.**

| **Baseline characteristics** | **Caucasians** | | | **East Asians** | | | |
| --- | --- | --- | --- | --- | --- | --- | --- |
|  | **Mean** | **SEM** | **N** | **Mean** | **SEM** | **N** | **P-value** |
| **Protein (g)** | 89.5 | 6.8 | **18** | 92.4 | 7.5 | **14** | **0.785** |
| **Fat (g)** | 78.4 | 5.4 | **18** | 84.1 | 5.9 | **14** | **0.488** |
| **Carbohydrate (g)** | 235.9 | 19.8 | **18** | 215.5 | 17.0 | **14** | **0.456** |
| **Energy intake (EI) (kcal)** | 1994.8 | 76.8 | **18** | 1974.6 | 81.3 | **14** | **0.859** |
| **Energy intake (EI) (kJ)** | 8362.7 | 322.4 | **18** | 8261.9 | 340.3 | **14** | **0.833** |
| **Total Saturates (g)** | 25.9 | 2.1 | **18** | 27.5 | 2.0 | **14** | **0.593** |
| **Total monounsaturate (g)** | 28.3 | 3.1 | **18** | 31.3 | 2.9 | **14** | **0.493** |
| **Total polyunsaturate (g)** | 13.3 | 1.3 | **18** | 17.3 | 1.9 | **14** | **0.088** |
| **Cholesterol (mg)** | 365.6 | 44.5 | **18** | 385.2 | 54.7 | **14** | **0.781** |
| **Total trans fatty acid (g)** | 1.4 | 0.3 | **18** | 1.4 | 0.2 | **14** | **0.882** |
| **Total n-3 fatty acid (g)** | 0.85 | 0.4 | **18** | 0.88 | 0.2 | **14** | **0.948** |
| **Total n-6 fatty acid (g)** | 3.9 | 1.2 | **18** | 6.8 | 1.6 | **14** | **0.150** |
| **Sodium (mg)** | 2326.5 | 187.0 | **18** | 2300.5 | 214.8 | **14** | **0.928** |
| ***Data are presented as mean ± SEM; N= sample size.*** | | | | | | | |

Table S2b. Main foods contributing to macronutrient intake and eating out habits reported by in participants

| **Top foods contributing to main macronutrients** | Caucasians | East Asians |
| --- | --- | --- |
| **Carbohydrates** | Breakfast cereals, Potatoes, pasta, white bread, | Rice, noodles, brown bread |
| **Protein** | Processed meat, chicken fresh meat, Egg, Milk and dairy products | Fresh meat, Processed meat, Egg, Fish, soybean products |
| **Fat** | Processed foods, vegetable oils, butter | Vegetable oils, Ghee |
|  |  |  |
| **Eating out on a weekly basis** | 5 | 7-10 |

**Table S2c.Adherence to the Mediterranean dietary guidelines as assessed by the PREDIMED score, and proportion of participants adhering to the different components of the score.**

|  | **Caucasian** | East **Asian** |
| --- | --- | --- |
| **Predimed score** | **Mean**± **SEM**  6.5 ± 1.8 | **Mean**± SEM  7.2 ± 1.8 |
|  |  |  |
|  | **%** | **%** |
| **(1) Used olive oil as main fat for cooking in diet (Yes)** | 0 | 0 |
| **(2) ≥4 tablespoons of olive oil a day** | 0 | 0 |
| **(3) ≥2 servings of vegetables a day (1 serving = 80g or 2 broccoli florets, three heaped tablespoons of cooked vegetables such as carrots or 3 celery sticks)** | 72.2 | 78.6 |
| **(4) ≥3 servings of fruit a day (1 serving =80g, or 1 banana, 2 kiwis, or 1 heaped tablespoon of sultanas)** | 50 | 28.6 |
| **(5) <1/week servings of red meat, hamburger, or meat products (1 serving =70g, or three slices of ham)** | 5.6 | 14.3 |
| **(6) <1/day servings of butter, margarine, or cream (1 serving =10g)** | 22.2 | 42.9 |
| **(7) <1/day servings of sweet or carbonated beverages (1 serving =150ml)** | 55.6 | 57.1 |
| **(8) ≥7/week glasses of wine (1 serving = 125ml)** | 11.1 | 0 |
| **(9) ≥3/week servings of legumes/pulses (1 serving =5 heaped tablespoons cooked)** | 44.4 | 50 |
| **(10) ≥3/week servings of fish or shellfish (1 serving =140g)** | 22.2 | 57.1 |
| **(11) <3/week servings of cakes, commercial sweets or pastries** | 33.3 | 35.7 |
| **(12) ≥3/week servings of unsalted nuts including peanuts (1 serving =40g)** | 16.6 | 28.6 |
| **(13) Preferentially consume chicken turkey or rabbit meat instead of veal, hamburger or sausage (Yes)** | 76.7 | 71.4 |
| **(14) ≥3/week consume a meal containing vegetables, pasta, rice, or other dishes seasoned with tomato based sauce** | 38.9 | 28.6 |

|  | **Model 1** | | | | | | **Model 2** | | | | |
| --- | --- | --- | --- | --- | --- | --- | --- | --- | --- | --- | --- |
| **Variable** | **N** | **Olive Oil** | | **Butter** | | **P** | **Olive Oil** | | **Butter** | | ***P*** |
|  |  | **Mean** | **SEM** | **Mean** | **SEM** |  | **LSM** | **SEM** | **LSM** | **SEM** |  |
|  | **All participants combined (n=32)** | | | | | | | | | | |
| **Grip strength mean (kg)** | 32 | 41.46 | 1.29 | 40.43 | 1.25 | 0.20 | 41.46 | 0.77 | 40.43 | 0.50 | 0.20 |
| **Grip strength maximum (kg)** | 32 | 43.69 | 1.37 | 42.94 | 1.28 | 0.35 | 43.69 | 0.78 | 42.94 | 0.63 | 0.37 |
| **Body fat (%)** | 32 | 16.45 | 1.00 | 16.93 | 0.96 | 0.26 | 16.45 | 0.41 | 16.93 | 0.37 | 0.27 |
| **Body fat (kg)** | 32 | 12.79 | 1.08 | 13.30 | 1.08 | 0.20 | 12.79 | 0.36 | 13.30 | 0.33 | 0.21 |
| **Lean (kg)** | 32 | 61.95 | 1.22 | 62.31 | 1.22 | 0.36 | 61.95 | 0.30 | 62.31 | 0.35 | 0.36 |

| **East Asians (n=14).** | | | | | | | | | | | |
| --- | --- | --- | --- | --- | --- | --- | --- | --- | --- | --- | --- |
| **Grip strength mean (kg)** | 14 | 41.693 | 1.209 | 40.879 | 1.396 | 0.511 | 41.693 | 1.028 | 40.879 | 0.633 | 0.446 |
| **Grip strength maximum (kg)** | 14 | 44.021 | 1.056 | 43.350 | 1.506 | 0.556 | 44.021 | 0.892 | 43.350 | 0.882 | 0.518 |
| **Body fat (%)** | 14 | 15.693 | 1.417 | 16.457 | 1.518 | 0.173 | 15.693 | 0.501 | 16.457 | 0.585 | 0.696 |
| **Body fat (kg)** | 14 | 11.786 | 1.426 | 12.579 | 1.616 | 0.180 | 11.786 | 0.421 | 12.579 | 0.488 | 0.181 |
| **Lean (kg)** | 14 | 60.664 | 1.908 | 60.457 | 1.838 | 0.561 | 60.664 | 0.390 | 60.457 | 0.371 | 0.738 |

| **Caucasians (n=18).** | | | | | | | | | | | |
| --- | --- | --- | --- | --- | --- | --- | --- | --- | --- | --- | --- |
| **Grip strength mean (kg)** | 20 | 41.278 | 2.13 | 40.10 | 1.972 | 0.277 | 41.278 | 1.139 | 40.083 | 0.727 | 0.306 |
| **Grip strength maximum (kg)** | 20 | 43.439 | 2.318 | 42.617 | 1.979 | 0.491 | 43.439 | 1.166 | 42.617 | 0.889 | 0.500 |
| **Body fat (%)** | 20 | 17.044 | 1.420 | 17.306 | 1.258 | 0.682 | 17.044 | 0.591 | 17.306 | 0.416 | 0.696 |
| **Body fat (kg)** | 20 | 13.567 | 1.583 | 13.861 | 1.472 | 0.602 | 13.567 | 0.541 | 13.861 | 0.327 | 0.622 |
| **Lean (kg)** | 20 | 63.012 | 1.572 | 63.835 | 1.574 | 0.510 | 63.012 | 0.279 | 63.835 | 0.569 | 0.074 |
| ***Model 1: Unadjusted***  ***Model 2: Estimated from repeated measures ANOVA of the Olive Oil and Butter adjustment with baseline, washout value as covariate. LSM = Least Squared Means (Marginal means).*** | | | | | | | | | | | |

**Table S3. Effects of extra virgin olive oil and butter on anthropometric data**

**Table S4. Effects of EVOO and Butter on blood pressure in Caucasians participants (n=18).**

|  | |  | **Model 1** | | | | | **Model 2** | | | | | |
| --- | --- | --- | --- | --- | --- | --- | --- | --- | --- | --- | --- | --- | --- |
| **Variable** | | **N** | **EVOO** | | **Butter** | | **P** | **EVOO** | | **Butter** | | **P** | |
|  |  |  | **LSM** | **SEM** | **LSM** | **SEM** |  | **LSM** | **SEM** | **LSM** | **SEM** |  | |
| Resting SBP (mmHg) | | 18 | 118.10 | 2.10 | 119.13 | 1.94 | 0.698 | 118.10 | 1.85 | 119.13 | 1.85 | 0.702 | |
| Resting DBP (mmHg) | | 18 | 68.22 | 1.54 | 68.39 | 1.58 | 0.936 | 68.22 | 1.55 | 68.39 | 1.84 | 0.941 | |
| Resting HR | | 18 | 69.71 | 2.01 | 72.92 | 2.01 | 0.228 | 69.71 | 2.13 | 72.92 | 2.29 | 0.273 | |
| **24-Hour ABP** | | | | | | | | | | | | |  |
|  | 24HA SBP (mmHg) | 18 | 120.21 | 1.50 | 123.14 | 1.97 | 0.256 | 120.21 | 1.42 | 123.14 | 2.14 | 0.269 | |
|  | 24HA DBP (mmHg) | 18 | 68.38 | 1.80 | 69.76 | 1.07 | 0.513 | 68.38 | 1.90 | 69.76 | 0.91 | 0.485 | |
|  | 24HA MAP (mmHg) | 18 | 86.26 | 1.64 | 87.52 | 1.21 | 0.531 | 86.26 | 1.71 | 87.52 | 1.03 | 0.502 | |
| **Daytime ABP** | |  | | | | | | | | | | |  |
|  | SBP (mmHg) | 18 | 127.23 | 2.04 | 129.98 | 1.61 | 0.312 | 127.23 | 2.14 | 129.98 | 1.82 | 0.378 | |
|  | DBP (mmHg) | 18 | 73.79 | 1.93 | 73.66 | 1.59 | 0.955 | 73.79 | 2.15 | 73.66 | 1.003 | 0.956 | |
|  | MAP (mmHg) | 18 | 92.22 | 1.98 | 92.13 | 1.32 | 0.971 | 92.22 | 2.09 | 92.13 | 0.999 | 0.973 | |
| **Nighttime ABP** | | | | | | | | | | | | |  |
|  | SBP (mmHg) | 18 | 112.37 | 2.28 | 116.59 | 2.28 | 0.151 | 112.37 | 2.18 | 116.59 | 1.90 | 0.197 | |
|  | DBP (mmHg) | 18 | 62.33 | 1.98 | 66.42 | 2.00 | 0.084 | 62.33 | 1.86 | 66.42 | 1.66 | **0.047** | |
|  | MAP (mmHg) | 18 | 79.47 | 1.83 | 83.40 | 2.09 | 0.09 | 79.47 | 1.71 | 83.40 | 1.50 | 0.06 | |
| ***Model 1: Estimated from repeated measures ANOVA of the EVOO and Butter adjustment with baseline and washout value as covariate.*** | | | | | | | | | | | | |  |
| ***Model 2 = Estimated from repeated measures ANOVA of the EVOO and Butter adjustment with baseline, intervention order sequence, washout, BMI, age, Mediterranean diet score, sodium intake and energy intake value as covariate.***  ***LSM = Least Squared Means (Marginal means).*** | | | | | | | | | | | | |  |

**Table S5. Effects of EVOO and Butter on blood pressure in East Asians participants (n=14).**

| **East Asians** | |  | **Model 1** | | | | | **Model 2** | | | | |  |
| --- | --- | --- | --- | --- | --- | --- | --- | --- | --- | --- | --- | --- | --- |
| **Variable** | | **N** | **EVOO** | | **Butter** | | **P** | **EVOO** | | **Butter** | | **P** |  |
|  |  |  | **LSM** | **SEM** | **LSM** | **SEM** |  | **LSM** | **SEM** | **LSM** | **SEM** |  |  |
| Resting SBP (mmHg) | | 14 | 115.28 | 2.52 | 117.75 | 1.87 | 0.179 | 115.28 | 2.51 | 117.75 | 1.48 | 0.251 |  |
| Resting DBP (mmHg) | | 14 | 70.77 | 1.99 | 68.33 | 2.20 | 0.319 | 70.77 | 2.02 | 68.33 | 2.42 | 0.377 |  |
| Resting HR | | 14 | 75.97 | 3.26 | 72.22 | 1.91 | 0.297 | 75.97 | 1.99 | 72.22 | 2.05 | 0.214 |  |
| **24-Hour ABP** | |  | | | | | | | | | | | |
|  | 24HA SBP (mmHg) | 14 | 114.72 | 1.847 | 120.63 | 1.40 | **0.012** | 114.72 | 2.19 | 120.63 | 1.14 | **0.026** |  |
|  | 24HA DBP (mmHg) | 14 | 68.70 | 1.671 | 71.57 | 2.05 | 0.254 | 68.70 | 1.33 | 71.57 | 1.05 | 0.107 |  |
|  | 24HA MAP (mmHg) | 14 | 84.46 | 1.298 | 88.30 | 1.73 | 0.051 | 84.46 | 1.33 | 88.30 | 1.04 | **0.038** |  |
| **Daytime ABP** | |  |  | | | | | | | | | | |
|  | SBP (mmHg) | 14 | 118.02 | 2.32 | 126.20 | 2.06 | **0.007** | 118.02 | 2.65 | 126.20 | 2.20 | **0.032** |  |
|  | DBP (mmHg) | 14 | 71.16 | 2.02 | 74.86 | 1.65 | 0.140 | 71.16 | 1.68 | 74.86 | 1.42 | 0.193 |  |
|  | MAP (mmHg) | 14 | 87.16 | 1.52 | 92.38 | 1.44 | **0.019** | 87.16 | 1.53 | 92.38 | 1.47 | 0.052 |  |
| **Nighttime ABP** | |  |  | | | | | | | | | | |
|  | SBP (mmHg) | 14 | 110.19 | 1.635 | 112.99 | 2.70 | 0.242 | 110.19 | 1.82 | 112.99 | 2.59 | 0.296 |  |
|  | DBP (mmHg) | 14 | 63.92 | 1.137 | 67.31 | 2.09 | 0.165 | 63.92 | 0.43 | 67.31 | 2.08 | 0.189 |  |
|  | MAP (mmHg) | 14 | 79.72 | 1.048 | 83.15 | 2.17 | 0.160 | 79.72 | 0.97 | 83.15 | 2.21 | 0.170 |  |
| ***Model 1: Estimated from repeated measures ANOVA of the EVOO and Butter adjustment with baseline and washout value as covariate.*** | | | | | | | | | | | | | |
| ***Model 2: Estimated from repeated measures ANOVA of the EVOO and Butter adjustment with baseline, washout, intervention order sequence, Mediterranean diet score, BMI, age, sodium intake and energy intake value as covariate.***  ***LSM = Least Squared Means (Marginal means).*** | | | | | | | | | | | | | |

**Table S6. Differences between daytime and nighttime blood pressures (Dipping) after interventions**

|  | **Dipping (%)**  **after EVOO** | | **Dipping (%)**  **After butter** | |
| --- | --- | --- | --- | --- |
| **All participants** |  | |  | |
| **Daytime SBP - Nighttime SBP** | 9.26% | | 10.38% | |
|  |  | |  | |
| **Daytime DBP - Nighttime DBP** | 12.89% | | 9.95% | |
|  |  | |  | |
|  |  | |  | |
| **Caucasians participants** |  | |  |  |
| **Daytime SBP - Nighttime SBP** | 11.68% | | 10.30% |  |
|  |  | |  |  |
| **Daytime DBP - Nighttime DBP** | 15.53% | | 9.82% |  |
|  |  | |  |  |
|  |  | |  |  |
| **East Asians participants** |  | |  |  |
| **Daytime SBP - Nighttime SBP** | 6.63% | | 10.5% |  |
|  |  | |  |  |
| **Daytime DBP - Nighttime DBP** | 10.17% | | 10.09% |  |
|  | |  |  | |

**Table S7. Effects of EVOO on blood-borne biomarkers in Caucasians male participants (n=18).**

|  |  | **Model 1** | | | | | **Model 2** | | | | | |
| --- | --- | --- | --- | --- | --- | --- | --- | --- | --- | --- | --- | --- |
| **Variable** | **N** | **EVOO** | | **Butter** | | ***P*** | **EVOO** | | **Butter** | | ***P*** | |
|  |  | **LSM** | **SEM** | **LSM** | **SEM** |  | **LSM** | **SEM** | **LSM** | **SEM** |  | |
| **TC (mmol/L)** | **18** | 159.7 | 3.1 | 168.6 | 2.7 | 0.064 | 4.13 | 0.08 | 4.36 | 0.06 | 0.104 | |
| **HDL (mmol/L)** | **18** | 52.6 | 0.8 | 53.0 | 1.5 | 0.779 | 1.36 | 0.02 | 1.37 | 0.04 | 0.802 | |
| **LDL (mmol/L)** | **18** | 95.5 | 2.3 | 104.0 | 2.7 | **0.046** | 2.47 | 0.05 | 2.69 | 0.03 | **0.041** | |
| **Non-HDL (mmol/L)** | **18** | 107.5 | 3.1 | 117.6 | 3.5 | **0.048** | 2.78 | 0.08 | 3.04 | 0.10 | 0.130 | |
| **Triglycerides (mmol/L)** | **18** | 81.5 | 8.9 | 82.4 | 5.3 | 0.959 | 0.92 | 0.13 | 0.93 | 0.06 | 0.970 | |
|  | | | | | | | | | | | |  |
| ***Model 1: Estimated from repeated measures ANOVA of EVOO and butter adjustment with baseline and washout value as covariate.*** | | | | | | | | | | | |  |
| ***Model 2: Estimated from repeated measures ANOVA of EVOO and butter adjustment with baseline. intervention order sequence, BMI, age, Mediterranean diet score, energy intake, saturated fatty acids, PUFAs, MUFAs, trans fatty acids, and cholesterol value as covariate.***  ***LSM = Least Squared Means (Marginal means).*** | | | | | | | | | | | |  |

**Table S8. Effects of EVOO and butter on blood-borne biomarkers in men of East Asians (n=14).**

|  |  | **Model 1** | | | | | **Model 2** | | | | | |
| --- | --- | --- | --- | --- | --- | --- | --- | --- | --- | --- | --- | --- |
| **Variable** | **N** | **EVOO** | | **Butter** | | ***P**** | **EVOO** | | **Butter** | | ***P***** | |
|  |  | **LSM** | **SEM** | **LSM** | **SEM** |  | **LSM** | **SEM** | **LSM** | **SEM** |  | |
| **TC (mmol/L)** | **14** | 4.46 | 0.15 | 4.66 | 0.16 | **0.044** | 4.46 | 0.14 | 4.66 | 0.18 | 0.079 | |
| **HDL (mmol/L)** | **14** | 1.24 | 0.03 | 1.31 | 0.04 | 0.112 | 1.24 | 0.03 | 1.31 | 0.05 | 0.354 | |
| **LDL (mmol/L)** | **14** | 2.49 | 0.07 | 2.62 | 0.08 | 0.173 | 2.49 | 0.07 | 2.62 | 0.04 | 0.320 | |
| **Non-HDL (mmol/L)** | **14** | 3.13 | 0.10 | 3.20 | 0.09 | 0.610 | 3.13 | 0.02 | 3.20 | 0.02 | 0.164 | |
| **Triglycerides (mmol/L)** | **14** | 1.89 | 0.57 | 1.39 | 0.19 | 0.257 | 1.89 | 0.52 | 1.39 | 0.16 | 0.273 | |
| ***Model 1: Estimated from repeated measures ANOVA of EVOO and butter adjustment with baseline and washout value as covariate.***  ***Model 2: Estimated from repeated measures ANOVA of EVOO and butter adjustment with baseline, intervention order sequence, BMI, age, Mediterranean diet score, energy intake, saturated fatty acids, PUFAs, MUFAs, trans fatty acids and cholesterol value as covariate.***  ***LSM = Least Squared Means (Marginal means).*** | | | | | | | | | | | |  |

**Table S9. Correlation for EVOO 24-HourABP SBP difference, Daytime SBP difference, Night-time DBP difference and Night-time MBP difference for both Caucasians and East Asians (n=32 participants)**

|  | **24-HourABP SBP difference (mmHg)** | **24-HourABP Daytime SBP difference (mmHg)** | **24-HourABP Night-time DBP difference (mmHg)** | **24-Hour ABP Night-time MBP difference (mmHg)** |
| --- | --- | --- | --- | --- |
| **Baseline BMI** | r=0.075  p=0.685 | r=-0.178  p=0.330 | r=-0.107  p=0.567 | r=-0.160  p=0.390 |
| **Age** | r=0.031  p=0.864 | r=-0.059  p=0.748 | r=0.097  p=0.604 | r=0.037  p=0.842 |
| **Fat (g)** | r=0.224  p=0.217 | r=0.500**  *p=0.004* | r=0.172  p=0.355 | r=0.205  p=0.268 |
| **Sodium intake** | r=-0.218  p=0.231 | r=-0.111  p=0.545 | r=-0.362  *p=0.045* | r=-0.311  p=0.088 |
| **Energy intake (Kcal)** | r=-0.085  p=0.644 | r=0.320  p=0.074 | r=-0.231  p=0.212 | r=-0.209  p=0.260 |
| **** Correlation is significant at the 0.05 level (2-tailed).***  ***** Correlation is significant at the 0.01 level (2-tailed).*** | | | | |

**Table S10.** **Determination of fatty acids as the corresponding fatty acid methyl esters in olive oil and butter samples (n=3). Only fatty acids with a % higher than 0.3% and/or identified are reported.**

| **Fatty acid** |  | **Olive oil (%)** | **Butter (%)** |
| --- | --- | --- | --- |
| **Butyric** | C4:0 | - | 1.8 (± 0.1) |
| **Caproic** | C6:0 | - | 0.3 ((± 0.08) |
| **Caprylic** | C8:0 | - | - 1. (± 0.1) |
| **Capric** | C10:0 | - | 1.8 (± 0.1) |
| **Lauric** | C12:0 | - | 2.9 (±0.03) |
| **Myristic** | C14:0 | - | 10.9 (±0.07) |
| **Pentadecanoic** | C15:0 | - | 1.2 (±0.02) |
| **Palmitic** | C16:0 | 13.3 (± 0.1) | 31.0 (±0.12) |
| **Palmitoleic** | C16:1n-7 | 0.95 ((± 0.05) | - |
| **Heptadecanoic** | C17:0 | - | 0.9 (±0.07) |
| **Stearic** | C18:0 | 2.8 (± 0.1) | 14.5 (±0.12) |
| **Oleic** | C18:1n-9 | 72.6 (± 0.7) | 25.1 (±0.15) |
| **Linoleic** | C18:2n-6 | 6.8 (± 0.2) | 1.6 (±0.14) |
| **Vaccenic** | C18:1n-7 | 2.3 (± 0.1) | 0.9 ((±0.01) |
| **Linolenic** | C18:3n-3 | 0.3 ((± 0.01) | 0.6 (±0.06) |
|  |  |  |  |
| **Saturated** |  | 16.1 | 64.8 |
| **Monounsaturated** |  | 79.4 | 26 |
| **Polyunsaturated** |  | 7.2 | 2.2 |

**CONSORT 2010 Checklist of information to include when reporting a randomised trial.**

| **Section/Topic** | **Item No** | **Checklist item** | **Reported on page No** |
| --- | --- | --- | --- |
| **Title and abstract** | | | |
|  | 1a | Identification as a randomized trial in the title | **1** |
|  | 1b | Structured summary of trial design, methods, results, and conclusions (for specific guidance see CONSORT  for abstracts) | **2** |
| **Introduction** | | | |
| Background and objectives | 2a | Scientific background and explanation of rationale | **3** |
|  | 2b | Specific objectives or hypotheses | **3** |
| **Methods** | | | |
| Trial design | 3a | Description of trial design (such as parallel, factorial) including allocation ratio | **4** |
| Participants | 4a | Eligibility criteria for participants | **4** |
|  | 4b | Settings and locations where the data were collected | **4** |
| Interventions | 5 | The interventions for each group with sufficient details to allow replication, including how and when they were  actually administered | **4** |
| Outcomes | 6a | Completely defined pre-specified primary and secondary outcome measures, including how and when they  were assessed | **5** |
|  | 6b | Any changes to trial outcomes after the trial commenced, with reasons | **N/A** |
| Sample size | 7a | How sample size was determined | **4** |
|  | 7b | When applicable, explanation of any interim analyses and stopping guidelines | **N/A** |

| **Randomization** | | | |
| --- | --- | --- | --- |
| Sequence generation | 8a | Method used to generate the random allocation sequence | **4** |
|  | 8b | Type of randomization; details of any restriction (such as blocking and block size) | **4** |
| Allocation  concealment mechanism | 9 | Mechanism used to implement the random allocation sequence (such as sequentially numbered containers), describing any steps taken to conceal the sequence until interventions were assigned | **4** |
| Implementation | 10 | Who generated the random allocation sequence, who enrolled participants, and who assigned participants to  interventions | **4** |
| Blinding | 11a | If done, who was blinded after assignment to interventions (for example, participants, care providers, those  assessing outcomes) and how | **4** |
|  | 11b | If relevant, description of the similarity of interventions | **N/A** |
| Statistical methods | 12a | Statistical methods used to compare groups for primary and secondary outcomes | **5** |
|  | 12b | Methods for additional analyses, such as subgroup analyses and adjusted analyses | **5** |
| **Results** | | | |
| Participant flow (a diagram is strongly recommended) | 13a | For each group, the numbers of participants who were randomly assigned, received intended treatment, and  were analyzed for the primary outcome | **8** |
|  | 13b | For each group, losses and exclusions after randomization, together with reasons | **8** |
| Recruitment | 14a | Dates defining the periods of recruitment and follow-up |  |
|  | 14b | Why the trial ended or was stopped | **N/A** |
| Baseline data | 15 | A table showing baseline demographic and clinical characteristics for each group | **17** |
| Numbers analyzed | 16 | For each group, number of participants (denominator) included in each analysis and whether the analysis was  by original assigned groups | **18** |
| Outcomes and estimation | 17a | For each primary and secondary outcome, results for each group, and the estimated effect size and its  precision (such as 95% confidence interval) | **18-20** |
|  | 17b | For binary outcomes, presentation of both absolute and relative effect sizes is recommended |  |
| Ancillary analyses | 18 | Results of any other analyses performed, including subgroup analyses and adjusted analyses, distinguishing  pre-specified from exploratory | **Supplementary material** |
| Harms | 19 | All-important harms or unintended effects in each group (for specific guidance see CONSORT for harms) | **N/A** |
| **Discussion** | | | |
| Limitations | 20 | Trial limitations, addressing sources of potential bias, imprecision, and, if relevant, multiplicity of analyses | **11** |
| Generalizability | 21 | Generalizability (external validity, applicability) of the trial findings | **11** |
| Interpretation | 22 | Interpretation consistent with results, balancing benefits and harms, and considering other relevant evidence | **9-11** |
| **Other information** | | | |
| Registration | 23 | Registration number and name of trial registry | **2** |
| Protocol | 24 | Where the full trial protocol can be accessed, if available | **2** |
| Funding | 25 | Sources of funding and other support (such as supply of drugs), role of funders | **N/A** |
